# Cutaneous Clue to Clonal Evolution: Sweet’s Syndrome Heralding Hematologic Progression in Essential Thrombocythemia

**DOI:** 10.1155/crh/9919558

**Published:** 2026-09-02

**Authors:** Kavya Ronanki, Bibhant Shah, Prisla Maria Dalton, Nikhil Nagpal, Kartik Saini, Sushantika, Nilotpal Chowdhury, Uttam Kumar Nath

**Affiliations:** ^1^ Department of Medical Oncology and Hematology, All India Institute of Medical Sciences, Rishikesh, India, aiims.edu; ^2^ Department of Dermatology, All India Institute of Medical Sciences, Rishikesh, India, aiims.edu; ^3^ Department of Pathology, All India Institute of Medical Sciences, Rishikesh, India, aiims.edu

**Keywords:** cytogenetics, essential thrombocythemia, malignancy, Sweet’s syndrome

## Abstract

Sweet’s syndrome, or acute febrile neutrophilic dermatosis, is a rare inflammatory condition that may occur idiopathically or in association with malignancy, drugs, or systemic disease. Among malignancy‐associated cases, hematologic neoplasms predominate. Its bullous variant is uncommon and is more frequently linked to underlying hematologic disorders, where it may act as a paraneoplastic manifestation or an early marker of disease progression. We report a 54‐year‐old woman with long‐standing, low‐risk essential thrombocythemia, diagnosed in 2011 and maintained on hydroxyurea and aspirin with stable blood counts for over a decade. She presented with an acute onset of painful erythematous swellings progressing to tense bullae over the hands and feet, accompanied by intermittent fever. Histopathological examination of a skin biopsy demonstrated dense neutrophilic infiltration of the dermis without vasculitis, consistent with Sweet’s syndrome. Systemic corticosteroid therapy led to rapid clinical improvement, and hydroxyurea was discontinued. Given the known association between Sweet’s syndrome and hematologic disease progression, further evaluation was undertaken. Bone marrow examination and cytogenetic analysis revealed a complex karyotype, while next‐generation sequencing identified an acquired TP53 mutation along with a CALR mutation, indicating clonal evolution and progression to a high‐risk disease state. This case highlights bullous Sweet’s syndrome as a rare but important cutaneous marker of clonal evolution in essential thrombocythemia. New‐onset neutrophilic dermatoses in patients with previously indolent myeloproliferative neoplasms should prompt dermatologic evaluation and comprehensive hematologic reassessment, as early recognition may facilitate timely diagnosis of disease progression and guide management.

## 1. Introduction

Sweet’s syndrome, also known as acute febrile neutrophilic dermatosis, is a rare inflammatory disorder characterized by sudden onset of painful erythematous plaques or nodules primarily on the upper extremities and head and neck with neutrophilic infiltration of the dermis and accompanied by systemic symptoms like fever and arthralgias. It can be idiopathic, malignancy‐associated, or drug‐induced. Approximately 21% of patients with Sweet’s syndrome have an associated malignancy, most commonly hematological, most common being acute myeloid leukemia (AML) [1]. The condition may present as a paraneoplastic manifestation, or it may be the cutaneous harbinger of an underlying malignancy or its recurrence [2]. Myeloproliferative neoplasms (MPNs), including essential thrombocythemia (ET), have been linked with Sweet’s syndrome in rare cases, often reflecting underlying immune dysregulation or evolving disease biology.

ET is a chronic MPN that typically follows an indolent clinical course, with long‐term survival in most patients. However, a subset may undergo clonal evolution, marked by acquisition of high‐risk cytogenetic and molecular abnormalities, leading to disease progression or transformation. Cutaneous paraneoplastic manifestations may precede or accompany such evolution and can serve as early clinical indicators of underlying hematologic instability.

We present here a rare case of Sweet’s syndrome in a patient with low‐risk ET, stable on hydroxyurea for over a decade, now presenting with painful bullous lesions and fever. Further workup led to the identification of clonal evolution characterized by a complex karyotype and an acquired TP53 mutation This case highlights Sweet’s syndrome as a potential cutaneous marker of disease progression in ET and underscores the importance of early dermatologic and hematologic evaluation in such presentations.

## 2. Case Presentation

A 54‐year‐old previously healthy female diagnosed in 2011 with ET (IPSET‐low risk), a platelet count of 1505 × 10^9^/L, and a severe headache managed on hydroxyurea and aspirin is in regular follow‐up in our OPD. The patient was stable with normal blood counts on follow‐up. Two months prior to presentation, she developed painful erythematous swellings, which subsequently developed into tense fluid‐filled blisters over her right hand and left foot which later involved both hands and feet over the preceding week. Some of the blisters ruptured spontaneously, leaving behind erosions and ulcerated areas. This was followed by diffuse swelling of both hands. It was associated with intermittent fever documented to 38.3°C associated with chills and malaise for 10 days. No history of mucosal involvement, systemic joint pains, or bowel complaints was reported. There was no history of cough, urinary symptoms, or other systemic complaints in the recent weeks prior to the illness. There was no history of any new drug taken for any symptoms. On physical examination, Patient was hemodynamically stable, and there was no evidence of splenomegaly. Multiple well‐defined, tense, tender fluid‐filled bullae containing turbid to hemorrhagic fluid with few well‐defined, irregular ulcerated plaque with overlying necrotic slough present over the dorsum of left hand, extending to involve the palmar aspect of the hand with similar tense bullae present on the medial border of the left foot (Figures 1, 2, and 3). Surrounding lesional erythema and edema of the left hand were present. No lymphadenopathy or other areas of skin involvement were noted. In view of rapidly progressing skin lesions, dermatology consultation was sought. Based on clinical findings, differential diagnosis of sweets syndrome, neutrophilic eccrine hidradenitis and bullous pyoderma gangrenosum were kept.

**FIGURE 1:**
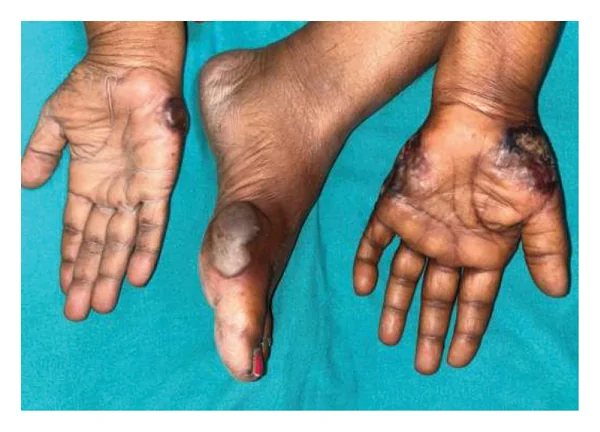
Clinical photograph showing multiple well‐defined, tense, fluid‐filled bullae over the palmar aspect of the left hand, with similar tense bullae in the medial border of the left foot.

**FIGURE 2:**
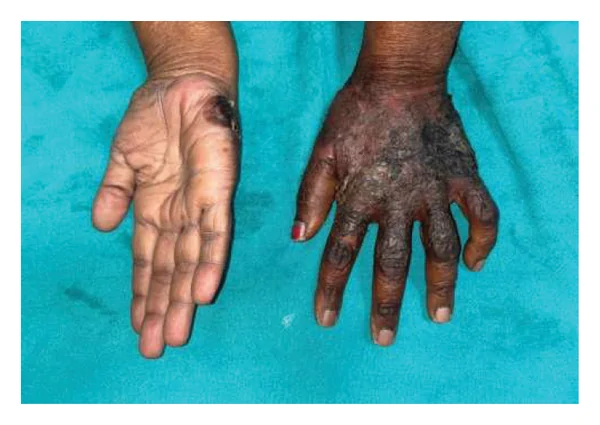
Clinical photograph demonstrating well‐defined ulcerated plaques with overlying necrotic slough involving the dorsum and extending to involve the medial aspect of the hand.

**FIGURE 3:**
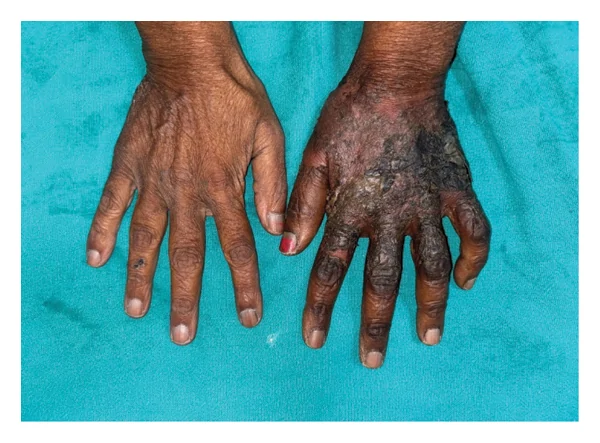
Multiple well‐defined, tense fluid‐filled bullae with few well‐defined ulcerated plaques with overlying necrotic slough present over the dorsum of the left hand.

Eccrine neutrophilic hidradenitis was considered due to acral distribution and fluid‐filled blisters which are classically seen in patients on chemotherapy but can occur otherwise. The bullous variant of bullous pyoderma gangrenosum can have a similar presentation, associated with hematological malignancies but not associated with systemic complaints. However, on laboratory workup, there is leucocytosis with 78% neutrophils and anaemia with a platelet count of 400–500 × 10^9^/L. A skin punch biopsy was performed under aseptic conditions which revealed dense neutrophilic infiltration consistent with Sweet’s syndrome (Figures 4, 5, and 6).

**FIGURE 4:**
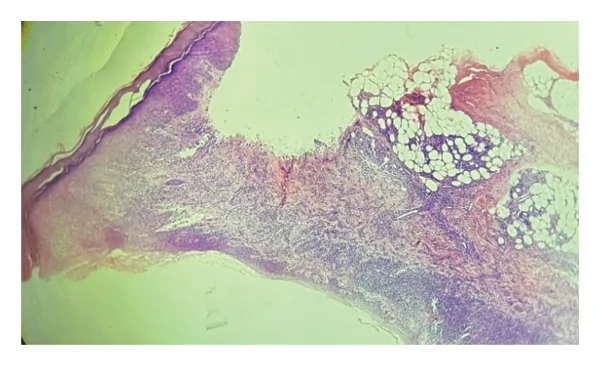
Scanner view demonstrating marked dermal edema with a dense neutrophilic infiltrate extending into the subcutaneous fat, without vasculitic changes.

**FIGURE 5:**
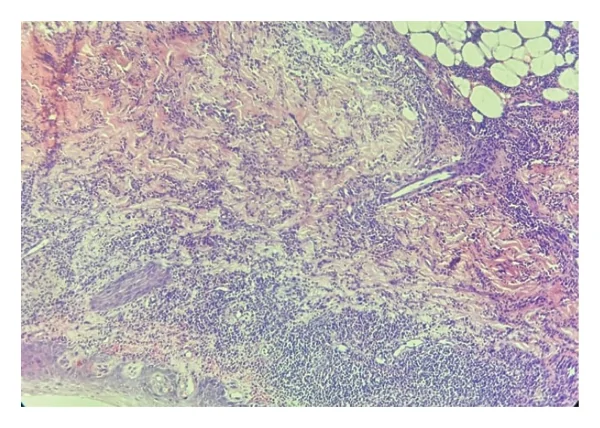
Histopathological section of skin biopsy showing dense neutrophilic infiltrate with marked dermal edema extending into the subcutaneous tissue, without evidence of vasculitis.

**FIGURE 6:**
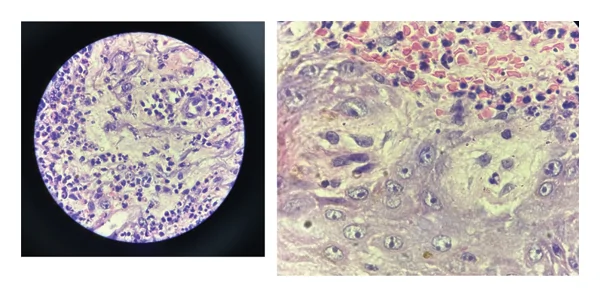
H&E‐stained section showing dense neutrophilic infiltrate in the upper dermis with dermal edema and absence of vasculitis, consistent with sweet’s syndrome.

The patient was started on Tab prednisolone 60 mg along with supportive treatment. Hydroxyurea was immediately withheld given its potential role in the cutaneous manifestations. Daily wound care with twice‐daily dressings was done. On follow‐up there was a drastic improvement of lesions and resolutions of hand edema and healing of the bullae within 10 days of therapy, fulfilling both major criteria and 4 minor criteria (fever + WBC> 8000, neutrophils > 70% + malignancy + rapid response to steroids) diagnostic criteria of Sweet’s syndrome [3]. As Sweet’s syndrome may signal disease recurrence or progression, The patient was planned for bone marrow studies including trephine biopsy to rule out disease progression which demonstrated chronic MPN consistent with ET. Conventional cytogenetic analysis revealed 44, XX, *t* (1,4) (q21; q31), −3, del (5) (q21), −7, add (6) (q36), −13, 18, add (18) (q23), +mar1, +mar2[cp10] (Figure 7). The presence of multiple structural abnormalities, monosomies, and marker chromosomes reflects significant clonal evolution in a disease typically characterized by genomic stability. Further molecular evaluation using next‐generation sequencing identified a TP53 missense mutation (p. Val272Met) with a variant allele frequency of 43.4%, along with a CALR frameshift mutation (p. Lys385AsnfsTer47) with a VAF of 61.5%. Detection of monosomy 7 and TP53 mutation signifies progression to a high‐risk disease phase. TP53 mutation is associated with high‐risk disease states in myeloid neoplasms and is strongly associated with genomic instability, increased risk of transformation to post‐ET myelofibrosis or acute leukemia, and inferior clinical outcomes, suggesting increased risk of progression to myelofibrosis or acute leukemia and a poorer prognosis warranting close monitoring and therapy. Although IPSET‐thrombosis classified the patient as low risk for thrombotic events, subsequent molecular and cytogenetic evaluation demonstrated high‐risk disease biology. Therapeutic options, including interferon, anagrelide, and clinical trial enrolment, including CALR‐directed therapies where available, were discussed. However, the patient declined further disease‐directed treatment at present and remains under close clinical, hematologic, and molecular surveillance.

**FIGURE 7:**
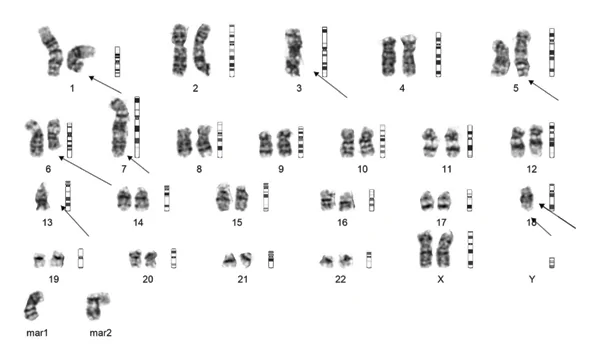
Karyotype demonstrating complex cytogenetic abnormalities in a patient with essential thrombocythemia. G‐banded chromosome analysis of 10 metaphases shows translocation between chromosomes 1 and 5 at bands q21 and q31 along with deletion of chromosome 6 at bands q21 and addition of extra genetic material on q arm of chromosomes 7 and 19 at bands 7q36 and 18q23 and loss of one copy of chromosomes 7, 13, and 18 and two marker chromosomes of unknown origin, respectively.

## 3. Discussion

Sweet’s syndrome, or acute febrile neutrophilic dermatosis, is an uncommon inflammatory disorder characterized by the abrupt onset of painful erythematous plaques or nodules, often accompanied by fever and leucocytosis [3]. Histologically, it is defined by a dense neutrophilic infiltrate in the upper dermis without evidence of vasculitis [4]. Although it can occur idiopathically, Sweet’s syndrome is increasingly recognized in association with malignancies, particularly hematologic neoplasms such as AML, myelodysplastic syndromes (MDS), and, more rarely, MPNs like ET [5].

Approximately 15%–21% of Sweet’s syndrome cases are associated with malignancy, and in some patients, it may precede the diagnosis of cancer or signal disease progression or recurrence [6]. The bullous variant, as seen in our patient, is particularly rare and more commonly linked to hematologic disorders. Its presentation may mimic infectious or autoimmune blistering diseases, often delaying diagnosis and management. In our case, the patient’s bullous lesions, systemic symptoms, and histopathological findings fulfilled both major and four minor criteria of Sweet’s syndrome, confirming the diagnosis [7].

Drug‐induced Sweet’s syndrome is another important differential in patients with MPNs, especially those receiving cytoreductive therapy such as hydroxyurea [8]. While hydroxyurea has been implicated in several reports as a causative agent for cutaneous reactions resembling Sweet’s syndrome, the presence of systemic symptoms, histologic features, and rapid clinical response to corticosteroids in our patient were more consistent with paraneoplastic Sweet’s syndrome rather than a drug‐induced aetiology. Nonetheless, hydroxyurea was prudently discontinued upon diagnosis.

Given the known association between Sweet’s syndrome and hematologic disease progression, further evaluation with bone marrow examination and cytogenetic analysis was undertaken. Notably, our patient demonstrated a complex karyotype, including *t* (1,4) (q21; q31), monosomies of chromosomes 3, 7, and 13, deletions and additions involving chromosomes 6, 7, and 18, and multiple marker chromosomes. These findings indicate clonal evolution and a transition to a high‐risk cytogenetic profile, which is highly unusual in otherwise low‐risk, chronic‐phase ET.

The presence of monosomy 7, in particular, is strongly associated with poor prognosis and an increased risk of transformation to acute leukemia [9]. The t(1,4) (q21; q31) translocation is rare but has been reported in association with PDGFRB rearrangements, which may confer sensitivity to tyrosine kinase inhibitors like imatinib, although further molecular characterization would be required to confirm such a target. The cumulative cytogenetic abnormalities in this case signify a marked shift from a stable disease phase to one at high risk for progression, underscoring the prognostic significance of paraneoplastic dermatologic manifestations.

The detection of a TP53 missense mutation (p.Val272Met) with a high variant allele frequency (43.4%) on next‐generation sequencing in our patient represents a critical molecular event underlying disease progression. TP53 mutations are uncommon in chronic‐phase ET but are well recognized in advanced or transforming myeloid neoplasms, including MDS and secondary AML [10]. Their presence is strongly associated with genomic instability, complex karyotypes, resistance to conventional therapy, and adverse prognosis. In MPNs, TP53 alterations often emerge as late events, reflecting clonal selection and evolution under disease pressure or therapeutic exposure [11].

In the present case, the coexistence of a CALR mutation with a newly acquired TP53 mutation and monosomy 7 suggests a shift from a previously stable ET clone to a genetically unstable, high‐risk clone. The high VAF of the TP53 mutation further supports its dominance within the hematopoietic compartment, consistent with disease acceleration toward a myelodysplastic phenotype [12]. Importantly, TP53‐mutated clones have been implicated in the pathogenesis of paraneoplastic inflammatory manifestations, including neutrophilic dermatoses, through dysregulated cytokine signaling and aberrant immune activation [13].

This case emphasizes the need for clinicians to maintain a high index of suspicion when patients with previously indolent MPNs develop atypical cutaneous lesions or systemic symptoms. Sweet’s syndrome, particularly in its bullous form, should prompt thorough hematologic reassessment, including bone marrow evaluation and early molecular and cytogenetic evaluation, as they may precede overt hematologic transformation. Early recognition and treatment with systemic corticosteroids often lead to rapid symptom resolution, but underlying disease progression may require modification of the long‐term management plan.

## 4. Conclusion

This case illustrates the importance of recognizing Sweet’s syndrome as a potential cutaneous marker of hematologic disease progression in patients with ET. The development of acute neutrophilic dermatosis in a patient with previously stable, low‐risk ET prompted further hematologic evaluation, which revealed clonal evolution characterized by a complex karyotype, monosomy 7, and an acquired TP53 mutation, signifying transformation to a high‐risk disease state. Early dermatologic assessment, prompt skin biopsy, and initiation of systemic corticosteroids resulted in rapid clinical improvement, while simultaneous hematologic reassessment allowed timely identification of disease progression. This case underscores the need for a multidisciplinary approach and highlights that new or atypical inflammatory cutaneous manifestations in MPNs should prompt early cytogenetic and molecular evaluation, as they may precede overt hematologic transformation and carry important prognostic and therapeutic implications.

## Funding

No external funding was received for this work.

## Disclosure

All authors have read and approved the final version of the manuscript.

## Ethics Statement

Ethical approval was not required for this single‐patient case report, in accordance with the institutional guidelines.

## Consent

Written informed consent was obtained from the patient for publication of the case report and accompanying clinical and pathological images.

## Conflicts of Interest

The authors declare no conflicts of interest.

## Patient Perspective

I was frightened when the blisters on my hands and feet appeared so suddenly and would not heal, and I could not understand why this was happening after so many stable years on my usual medication. I am relieved that the steroid treatment worked quickly and my skin healed, but I was anxious to learn that further tests showed my blood disease had changed. I am grateful to my doctors for explaining what was happening and for continuing to monitor me closely, and I feel reassured knowing my condition is being followed carefully even though I have chosen not to start additional treatment for now.

## Data Availability

The data supporting the findings of this case report are contained within the manuscript. Additional patient‐level data are not publicly available to protect patient privacy.
